# Insights into Endothelial Progenitor Cells: Origin, Classification, Potentials, and Prospects

**DOI:** 10.1155/2018/9847015

**Published:** 2018-11-18

**Authors:** H. Chopra, M. K. Hung, D. L. Kwong, C. F. Zhang, E. H. N. Pow

**Affiliations:** ^1^B.D.S., M.D.S. (Conservative Dentistry and Endodontics), Research Postgraduate Student, Discipline of Prosthodontics, Faculty of Dentistry, The University of Hong Kong, Pok Fu Lam, Hong Kong; ^2^BChinMed, Research Assistant, Department of Surgery, Queen Mary Hospital, The University of Hong Kong, Pok Fu Lam, Hong Kong; ^3^MBBS, MD, FRCR, FHKCR, FHKAM, Clinical Professor, Department of Clinical Oncology, Li Ka Shing Faculty of Medicine, The University of Hong Kong, Pok Fu Lam, Hong Kong; ^4^D.D.S, Ph.D. (Med), Clinical Professor, Discipline of Endodontics, Faculty of Dentistry, The University of Hong Kong, Pok Fu Lam, Hong Kong; ^5^B.D.S., M.D.S. (Prosthetic Dentistry), MRACDS (Pros), FRACDS, FCDHK (Pros), FHKAM (Dental Surgery), Clinical Associate Professor, Discipline of Prosthodontics, Faculty of Dentistry, The University of Hong Kong, Pok Fu Lam, Hong Kong

## Abstract

With the discovery of endothelial progenitor cells (EPCs) in the late 1990s, a paradigm shift in the concept of neoangiogenesis occurred. The identification of circulating EPCs in peripheral blood marked the beginning of a new era with enormous potential in the rapidly transforming regenerative field. Overwhelmed with the revelation, researchers across the globe focused on isolating, defining, and interpreting the role of EPCs in various physiological and pathological conditions. Consequently, controversies emerged regarding the isolation techniques and classification of EPCs. Nevertheless, the potential of using EPCs in tissue engineering as an angiogenic source has been extensively explored. Concomitantly, the impact of EPCs on various diseases, such as diabetes, cancer, and cardiovascular diseases, has been studied. Within the limitations of the current knowledge, this review attempts to delineate the concept of EPCs in a sequential manner from the speculative history to a definitive presence (origin, sources of EPCs, isolation, and identification) and significance of these EPCs. Additionally, this review is aimed at serving as a guide for investigators, identifying potential research gaps, and summarizing our current and future prospects regarding EPCs.

## 1. Introduction

Prevascularization is one of the critical approaches to enhance the success of tissue-engineered grafts [1]. A lack of vascular perfusion compromises the oxygen and nutrient supply as well as the disposal of wastes and toxins, leading to cell death, poor integration, and graft failure [2]. Therefore, neovascularization is currently considered the fourth pillar of the preexisting tissue engineering triad: stem cells, growth factors, and scaffold [3]. The term “haemangioblast” was proposed almost a century ago to describe the common origin of haematopoietic/endothelial progenitor cells [4]. However, the existence of haemangioblast was substantiated only two decades ago by Asahara and his colleagues [5], whom successfully isolated “endothelial progenitor cells” (EPCs) from the human peripheral blood. This discovery resulted in a mammoth global exploration of EPCs by researchers. Concurrently, controversies regarding the origin of EPCs, ambiguity in the phenotyping of EPCs, and nonstandardized isolation techniques have emerged besides difficulties in the isolation of EPCs.

This review is aimed at providing comprehensive insight into endothelial cells (ECs) from basic terminologies to its origin, the source of EPCs, EPC isolation techniques, the impact of EPCs on various therapies, and future prospects. Furthermore, this review will discuss the potentially unaddressed areas where research could have a substantial influence on the domain of neovascularization, and in turn, EPCs.

## 2. What Is Neovascularization?

Most of the tissue engineering studies and modern disease interventions are based on the augmentation or inhibition of angiogenesis. For example, in tissue-engineered grafts, amplification of angiogenesis is desired, whereas in tumours, suppression of angiogenesis is considered as an essential therapeutic application. However, the word “angiogenesis” is a misnomer, as it is a generic term that does not apply to all cases. Therefore, it is pragmatic to clarify the mechanism of blood vessel formation. Angiogenesis is defined as the formation of new capillaries from preexisting vessels [6]. De novo blood vessel formation during embryonic development is called “vasculogenesis,” while “postnatal vasculogenesis” describes new blood vessel formation in adults [7]. On the other hand, “arteriogenesis” is defined as the maturation and formation of larger-diameter arteries from preexisting capillaries or collateral arteries [8]. The novel term “neovascularization” has been suggested to embody all types of vessel formation in adults [9].

## 3. Endothelial Progenitor Cells

**Figure 1:**
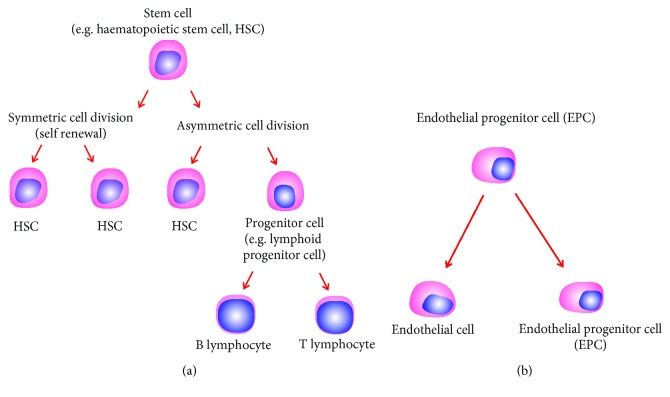
Difference between stem cells and progenitor cells.

Stem cells have been traditionally characterized based on three properties: self-renewability, clonogenicity, and plasticity (differentiation capacity). In sharp contrast, progenitor cells lack self-renewability. EPCs are unique, as they are distinctly different from progenitors but are similar to stem cells with a similar triad of self-renewability, clonogenicity, and differentiation capacity (Figure 1).

Further, EPCs are mostly unipotent stem cells which can uptake acetylated low-density lipoproteins (acLDL), bind with *Ulex europaeus* agglutinin-1 (UEA-1), and take part in neovascularization through either paracrine or autocrine mechanisms. To date, two different types of EPCs have been recognized and are described according to their morphologies, time of appearance, and expression of proteins. Both types of EPCs, along with other ECs, will be discussed later in the section for better insight.

## 4. Origin of Endothelial Cells (ECs)

It has been contemplated that during embryogenesis, a special type of cell called “haemangioblast” is the precursor of both endothelial and haematopoietic cell lineages. The term “haemangioblast” was coined by Murray [4] and is different from “angioblast,” as initially suggested by Sabin [10]. Accordingly, the term “angioblast” should be restricted to the vessels only, i.e., to the endothelium, whereas the term “haemangioblast” refers to a solid mass of cells that gives rise to both endothelium and blood cells. The hypothesis that ECs originate from haemangioblast is based on the close developmental association of the haematopoietic and endothelial lineages within blood islands [4, 10, 11]. However, these studies failed to reach a definite conclusion due to the complexities in acquiring chick embryos before the development of blood islands and the negligible number of cells present during this stage.

Nevertheless, rapid advances in medical field by the end of the twentieth century spurred the studies with embryonic stem cell differentiation models (ESCDM) [12–14], genetics, and newer animal models [15] and reported a spatiotemporal association between haematopoietic and endothelial lineages during earlier stages of life.

The earliest ESCDM was a mouse wherein an embryonic stem cell (ESC) line isolated from a mouse [16, 17] laid the foundation for studying mammalian developmental biology [18]. Differentiation of these ESCs has distinct advantages for examining the sequelae of initial cellular and molecular changes at the onset [12]. For example, mESC differentiation studies identified blast colony-forming cells (BL-CFCs), a type of progenitor cell that is the precursor of both haematopoietic cells and ECs [13]. Further, kinetic analysis has demonstrated that BL-CFCs represent the establishment of primitive erythroid and other lineage-restricted precursors [19]. Additionally, embryoid bodies (EBs), which are differentiated ESCs, have also indicated that multiple haematopoietic lineages can originate from ESCs in culture [20]. With these intriguing results and persistent efforts, hESCs were successfully isolated after almost two decades and their *in vitro* differentiation also leads to both haematopoietic and EC lineages [21, 22]. However, the first evidence that human haemangioblasts are comparable to mouse haemangioblasts was only recently reported in a study showing that human BL-CFCs, similar to mouse BL-CFCs, have both haematopoietic and vascular potential [23].

In conjunction with the cell culture studies, the study of various receptors and transcription factors and biochemical analyses of regulatory factors have provided detailed insight into the hypothesis that ECs originate from haemangioblasts. Receptor tyrosine kinases (RTKs) are considered as key regulators of developmental processes. Foetal liver kinase 1 (FLK-1) (also known as KDR) is an RTK that has been identified in primitive and more mature haematopoietic cells as well as in a wide variety of nonhaematopoietic tissues [24]. Functional analysis of FLK-1 revealed that FLK-1 is expressed in blood islands in mouse embryos and is therefore pivotal in regulating both vasculogenesis and angiogenesis [25]. In a knockdown experiment, *FLK-1* gene-deficient mouse embryos failed to generate blood islands as well as endothelial and haematopoietic cells [26]. Cell sorting further confirmed that FLK-1^+^ cells represent the earliest precursors of embryonic haematopoiesis [27, 28], whereas FLK-1^+^VE cadherin*^+^* cells signify a diverging point of haematopoietic and endothelial cell lineages [28]. Vascular endothelial growth factor (VEGF) is a potent angiogenic factor whose interaction with FLK-1 is responsible for the formation of BL-CFCs [13, 19]. TIE-2, an endothelial cell marker, is not expressed in the mesoderm of the primitive streak but is present in the haemangioblast [29]. Therefore, expression of TIE-2 substantiates the presence of an intermediate stage that gives rise to both haemogenic endothelium (HE) and angioblasts [30].

Another receptor that is expressed at the onset of primitive and definitive haematopoiesis (PH and DH) is CD41 [31–33]. CD41 is a platelet glycoprotein receptor that is required for normal platelet haemostatic function [34]. Although CD41 was previously thought to be lineage restricted, various studies have demonstrated differential expression of CD41, indicating a dynamic regulation of CD41 in haematopoietic development [31–33]. The phenotype of HE was found to be C-KIT^+^TIE-2^+^CD41*^−^* of which two-third contributes to ECs with the same gene expression while the rest of the population differentiates to hematopoietic precursors, i.e., haematopoietic stem cells (HSCs). This transition from HE to haematopoietic cells does not occur by asymmetric cell division but by a unique method referred to as endothelial to haematopoietic cell transition (EHT) [35, 36]. On the other hand, PH is speculated to evolve from angioblasts with a C-KIT^+^TIE-2^+^CD41^+^ signature [30].

There are also myriad TFs that play a significant role in haematopoiesis. Of these, TFs from the GATA family and RUNX-1 are most commonly involved. Some TFs have distinct roles in either PH or DH, whereas some are imperative for both. For example, it has been reported that GATA-1^−^ progenitor cells give rise to PH, whereas the GATA-1^+^ subpopulation differentiates into VE cadherin^+^ cells that give rise to both endothelial and haematopoietic cells [37]. On the other hand, RUNX-1 is essential for DH but does not affect PH [38–40]. However, SCL/TAL-1 (stem cell leukaemia/T cell acute lymphocytic leukaemia 1) and *LMO-2* (LIM domain TF) are necessary for both PH and DH as well as vascular remodelling [41, 42]. Functional studies have shown that forced expression of SCL mRNA in zebrafish embryos resulted in the development of both haematopoietic and endothelial precursors, suggesting a role for the *SCL* gene in haemangioblast formation [43]. Furthermore, both SCL/TAL-1 and LMO-2 act synergistically to stimulate the formation of haemangioblasts [44, 45], which in the absence of GATA-1 differentiate into ECs only [44].

Significant results from chick and mouse embryos encouraged researchers to explore other animal models. As a result, haemangioblasts were also identified in Drosophila [46] and zebrafish [47]. Zebrafish has a distinct advantage as a vertebrate which makes it a perfect model for genetic analysis and experimental embryology [48–50]. Further, as zebrafish embryos are transparent, tracing techniques emerged for mapping embryonic development until cardiac development [51–53]. In fact, by fate mapping in zebrafish, haemangioblasts and cardiomyocytes were found to work antagonistically [54].

**Figure 2:**
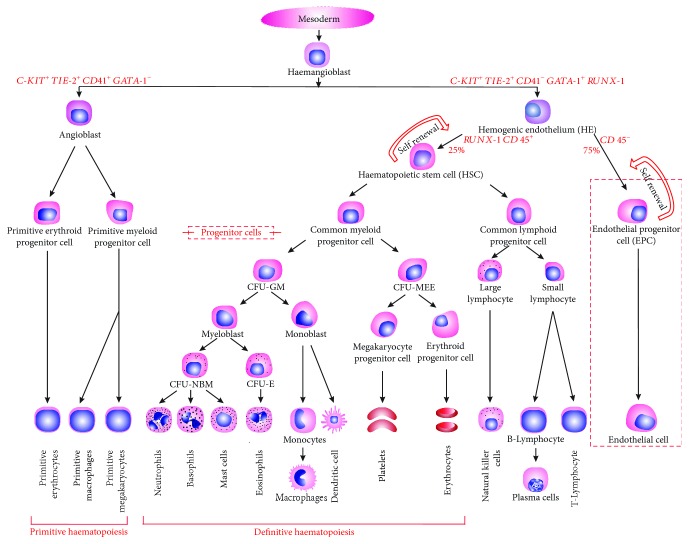
Origin of ECs from haemangioblast: haematopoiesis.

Although the existence of haemangioblasts was proposed a century ago, it is still a matter of extensive research and debate. Despite these controversies, the above-discussed findings support the existence of haemangioblasts. In summary, HE and angioblasts are intermediate stages of haemangioblasts. Angioblasts give rise to the first wave of PH, whereas HE gives rise to the second wave of DH. A detailed model is shown in Figure 2, explaining the origin of EPCs as well as the blood cell hierarchy.

## 5. Types of EPCs

### 5.1. Based on the Source

**Figure 3:**
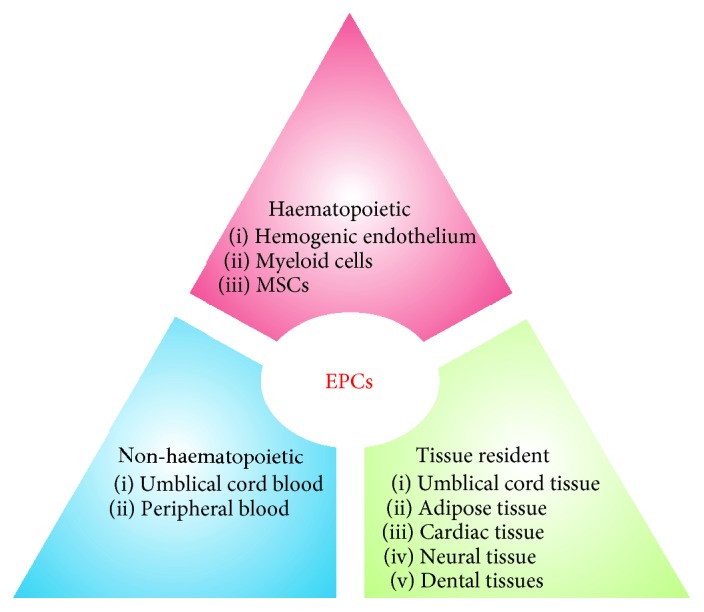
Different sources of EPCs.

As discussed above, EPCs shared a common precursor with other lineages. Therefore, it is plausible that these EPCs can be isolated/transdifferentiated from various sources and, hence, are sharing similar phenotypic characteristics. Therefore, we are providing a comprehensive outline of different sources of EPCs (Figure 3).

#### 5.1.1. Haematopoietic EPCs

*(1) Bone Marrow-Derived Endothelial Cells (BMECs).* Bone marrow is a complex microenvironment consisting of different cells (Figure 2). BMECs reside in close association with various cell types, which makes the isolation of ECs very challenging. BMECs were first isolated from rat [55] or murine [56] bone marrow by the density centrifugation method or differential phagocytosis of magnetic beads, respectively. On the other hand, human BMECs were isolated either directly from bone marrow aspirate or indirectly (enzymatic digestion of spicules present in the bone marrow followed by culture of cells) using the magnetic-activated cell sorting (MACS) assay via selective binding of ECs to UEA-1 [57, 58]. Later, the isolation of human BMECs was simplified by using mononuclear cells (MNCs) obtained by density centrifugation of bone marrow aspirate and then subjecting MNCs to either MACS (using UEA-1-, CD146-, and BNH9-coated beads) or fluorescence-activated cell sorting (FACS; using CD146 or BNH9). It was found that ECs were best obtained with FACS and constituted 0.05% of MNCs [59]. The isolated ECs in variously mentioned studies were characterized by immunofluorescence staining, such as factor VIII, vWF (von Willebrand factor), CD34, CD31, E-selectin (CD62E), ICAM-1 (CD54), and VCAM-1 (CD106) [55–58]; biochemical analysis where ECs were found to be alkaline phosphatase negative but were acid phosphatase positive [56]; ultrastructural identification of Weibel-Palade bodies by electron microscopy [55, 57, 58]; positive lectin binding (UEA-1) [56]; and analysis of surface markers by flow cytometry, such as vWF, CD34, CD31, CD14, ICAM-1, and VCAM-1 [58, 59]. Further, examination of surface morphology revealed different types of cells, for example, spindle-shaped [57–59], round shaped [56, 57], or cobblestone-shaped cells [58]. However, these cell shapes and indeed ECs were not well defined until ECs were isolated from peripheral blood [5], which portrays how a momentous research leads to an escalated renaissance in haematology.

There has been a radical shift of focus from BMECs to ECs in blood because peripheral blood ECs were stated to originate from BMECs [60, 61], although it is controversial and disputed at times [62]. In addition, withdrawing blood is a relatively noninvasive procedure. The movement of cells from bone marrow to peripheral blood that contributes to circulating endothelial progenitors (CEPs) is referred to as mobilization. As a result, ECs in the peripheral blood serve as the biomarker of various pathophysiological conditions, whereas BMECs represent the hot target zone. For example, ablating bone marrow endothelial progenitors not only does impair tumour growth associated with reduced vascularization [63] but also endorses the notion that ECs originate from bone marrow. However, there are other studies which substantiate that bone marrow-derived ECs do not contribute to vascular endothelium and tumour growth [64]. Nevertheless, despite controversies, BMECs still play a major role in neovascularization.

*(2) Myeloid Cells.* Myeloid cells are CD14^+^, and EPCs are CD14^−^. However, when CD14^+^ monocytes were isolated and grown under endothelial conditions for four weeks, there was an 80% reduction in CD14 expression with a significant increase in the expression of endothelial cell markers, such as vWf, VE, and eNOS (endothelial nitric oxide synthase/endothelial protein kinase A). Furthermore, these stimulated cells also developed cord- and tubular-like structures in vitro. Therefore, monocytes or cells with the CD14^+^ phenotype can also acquire an endothelial phenotype under angiogenic conditions [65]. Also, when EPCs derived from induced CD14^−^ mononuclear cells were implanted into ischaemic hind limbs, immediate neovascularization was observed but neovascularization did not occur when uninduced CD14^+^ cells or macrophages and dendritic cells derived from CD14^+^ cells were introduced [66].

*(3) Mesenchymal Stem Cells (MSCs).* As discussed above, adult bone marrow is a heterogenous mixture of haematopoietic as well as mesenchymal stem cells. Friedenstein and coworkers were the first to isolate colony-forming unit fibroblasts (CFU-Fs) from bone marrow [67]. The name CFU-F has been progressively replaced by various indistinct terms, such as marrow stromal fibroblasts (MSF), marrow stromal stem cells [68], mesenchymal adult progenitor cell (MAPC) [69], or the now widely accepted term mesenchymal stem cells (MSCs). These MSCs were isolated by two techniques: either by density centrifugation [70] or by isolating CD45^−^/glycophorin A^−^/TERR119^−^ cells from bone marrow cells [69, 71]. Flow cytometric analysis revealed that these MSCs were positive for CD29, CD71, CD73 (SH3), CD90, CD105 (SH2), CD106, CD144, CD120a, and CD 124 [69–72], while they were negative for CD34, CD31, VEGFR2, CD62E, vWF, VE-cadherin, VCAM-1, and ICAM-1 [69–72]. CD44 was present in some studies [70, 72], whereas it was absent in others [69].

When these MSCs were grown under endothelial conditions, they acquired characteristics of ECs and were found to be positive for VEGFR2, vWF, and VE-cadherin [69, 71, 72]. It is noteworthy that CD31 was expressed late [69, 72]. Also, it was found that these differentiated ECs contributed to neovascularization in tumour models [69, 71] and wound healing models [69]. Moreover, when undifferentiated MSCs were injected, they not only contributed to increased vascularity [69, 73] but also augmented cardiac function in chronic ischaemic models suggesting that transdifferentiation of MSCs to ECs is mediated through a paracrine mechanism [74].

#### 5.1.2. Nonhaematopoietic EPCs

*(1) Peripheral Blood.* Blood vessels are lined by the endothelium which was initially perceived to be a fixed structure having limited or no self-renewal ability. However, the hypothesis changed with the earliest study providing evidence that there are certain cells present in the blood which are responsible for endothelial turnover [75]. Thereafter, the levels of ECs or EC remnants were reported to be raised in the blood of patients with cardiovascular diseases [76, 77] and cancer [78]. At that time, the method of EC identification was crude using either cytologic staining of cell smears from leukocyte concentrate [76, 78] or morphologic recognition of EC-like “carcasses” in platelet-rich plasma [77]. The earliest method to quantify ECs was based on the separation of ECs from the whole blood based by density gradient sedimentation. However, this method was not EC specific [79], and therefore, another method was followed which used indirect immunofluorescence with the CLB-HEC 19 antibody to specifically identify EC cells [80]. In this study, it was reported that the minimal detectable concentration of CECs was 0.06 cells/mL of whole blood [80].

However, it was not until the end of the twentieth century when Asahara et al. revolutionized haematopoiesis and neovascularization by isolating and culturing endothelial cells from the peripheral blood [5]. These ECs have “spindle-shaped” morphology and were characterized by various markers, an ability to uptake acLDL and an ability to bind UEA-1. These “spindle-shaped” ECs were later termed early EPCs (eEPCs) [81] or circulating angiogenic cells (CACs) [82]. However, if the MNCs are cultured for a longer period, such as >2 weeks, ECs with a “cobblestone” morphology appear and are referred to as outgrowth endothelial cells (OECs) [83], late EPCs (lEPCs) [81], or endothelial colony-forming cells (ECFCs) [84]. Collectively, these cells are termed as circulating endothelial progenitors (CEPs). After the experiments by Asahara and his coworkers [5], numerous studies were conducted to isolate, classify, and define these eEPCs and lEPCs. We have enumerated the differences between eEPCs and lEPCs in Table 1.

Although there is no accord between the phenotype of CECs and CEPs, emerging evidence from the plethora of studies indicate that CECs are distinctly different from CEPs. CECs are mature cells that are not culturable and might consist of two types of the population of cells: firstly, the majority of the cells that are sloughed off from the vessel wall either normally or abnormally and secondly, cells that are matured from lEPCs or in various stages of maturation from CEPs that may or may not reside in the vessel wall. In normal cases, CECs as well as CEPs are extremely low. However, their concentration is influenced by various exogenous factors, endogenous factors, and pathological conditions which are discussed elaborately in this review. For example, CECs in healthy subjects were <3 cells/mL of whole blood. However, in patients with sickle cell anaemia, their concentration increases to 5–10-fold [85]. Similarly, lEPCs were found to be in between 0.05 and 0.2 cells/mL [84]; however, their concentration increases markedly after exercise [86]. Therefore, it is quite obvious that CECs originated from the vessel wall (e.g., conditions related to endothelial dysfunction) and represent biomarkers for vascular injury (e.g., CVD), whereas CEPs originate from bone marrow with conditions that will either stimulate bone marrow (such as tissue ischaemia in exercise) or suppress bone marrow (e.g., diabetes) and may (e.g., myocardial infarction) or may not (e.g., tumour) contribute to vascular repair.

**Figure 4:**
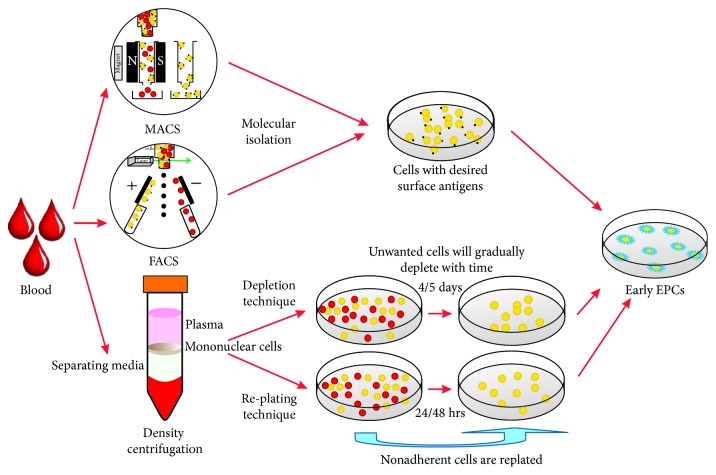
Isolation of EPCs by various techniques.

Nevertheless, the isolation of these ECs can be summarized into three basic techniques, which were later modified by various researchers (Figure 4).

*(1.1) Molecular Isolation*. In this technique, cells are identified based on their expression of cell surface markers. There are two methods that facilitate molecular recognition; one is MACS which employs the use of magnetic beads coated with the antibody/protein of choice, and another is FACS, which works on the principle of excitation and emission of fluorochromes bonded to the antibody/protein. Isolation of BMECs by MACS using UEA-1 was the earliest evidence in the literature of ECs [57, 58]. In fact, MACS was also used in the landmark study to isolate CD34^+^ cells from human peripheral blood with the aim to identify putative ECs [5]. When these CD34^+^cells were plated on FN-coated dishes, they became spindle shaped within three days. However, when both CD34^+^ cells and CD34^−^ cells were cocultured, clusters of round cells appeared centrally with spindle-shaped cells at the periphery. This morphology represented reminiscent of the blood island-like groups typically found in the developing embryonic yolk sac [87]. Additionally, when these CD34^+^ cells were injected into rabbits or ischaemic mouse hind limbs, DiI-labelled CD34^+^ cells were localized exclusively at the neovascular zones of the ischaemic limb [5]. The assay can also now be performed using a commercially available kit (EndoCult; STEMCELL Technologies, Vancouver, BC, Canada).

On the other hand, FACS is relatively technique sensitive but with rapid advancement in technology; instead of MACS, FACS has not only gained significant attention but is now the mainstay to isolate, classify, and analyse CEPs as well as CECs [88–90] because of its versatility and ease in obtaining a high percentage of pure populations.

*(1.2) Depletion Technique*. The depletion technique involves plating MNCs on FN-coated dishes for approximately four days. The nonadherent cells are then removed by washing with PBS, leaving MNCs on the dish. The four-day period is selected because the unwanted platelets, red blood cells, or monocytes are gradually depleted over this period. The number of days is, however, not fixed and has been modified by various researchers. Spindle-shaped cells, referred to as eEPCs, will appear after 6-7 days of culture [81], whereas “cobblestone” cells, referred to as lEPCs, will appear after four weeks in culture [81]. This procedure is not only used widely to isolate and characterize EPCs but has also been modified by various researchers [81, 82, 91, 92].

*(1.3) Replating Technique*. The fundamental principle of the replating method is to replate the nonadherent cells after plating the MNCs. The rationale for preplating the MNCs is to remove any monocytes, macrophages, or circulating mature ECs that might be present in the MNC sample [93]. The nonadherent cells were recovered either after 24 hours [94] or after 48 hours [95] and then replated and assessed. The later assay has been commercialized and is referred to as colony-forming unit Hill assay. CFU-Hill assay has demonstrated a significant inverse correlation between the circulating CFU-Hill concentration and Framingham cardiovascular risk score in human subjects [95]. However, the use of this technique for the isolation of EPCs has not garnered significant attention as it has resulted in mixed results.

*(2) Umbilical Cord Blood (UCB) EPCs*. Human cord blood is a rich source of HPs [96]. EPCs have been successfully isolated from UCB [84, 97]. In fact, when the same volumes of UCB and peripheral blood were taken for isolating EPCs, not only the EC colonies appeared earlier in UCB but also these colonies were larger in size as well as 15 times more than that found in adult peripheral blood [84]. Additionally, the plasticity and telomerase activity of UCB-derived EPCs are also much higher than those of peripheral blood-derived EPCs. Moreover, when UCB-derived EPCs were transplanted in the ischaemic hind limb of immunodeficient nude rats, it promoted limb recovery by neovascularization of ischaemic hind limbs [97].

#### 5.1.3. Tissue-Resident EPCs

*(1) Umbilical Cord*. In the Wharton's jelly (the connective tissue within the umbilical cord), abundant cells that exhibit MSC markers (SH2 and SH3) but not markers of haematopoietic differentiation (CD34 and CD45) were found and they were named umbilical cord stem cells (UCSCs) [98]. Furthermore, MSC-like cells were also isolated from the subendothelial layer of the umbilical cord vein [99]. When these UCSCs were subjected to endothelial conditions, they differentiated into ECs with a phenotype and function-like lEPCs. Additionally, when these EPCs were transplanted in murine ischaemic hind limbs, they promoted neovascularization [100].

*(2) Adipose Tissue*. Human adipose tissue also contains multipotent stem cells that can be easily harvested. Processed lipoaspirate contains cells that show multidifferentiation potential similar to MSCs but have a different phenotypic characterization [101, 102]. This unique population of cells distinct from MSCs is called adipose-derived stem cells (ADSCs) [101, 102]. The CD34^+^CD31^−^ ADSCs can differentiate into ECs and augment postnatal neovascularization [103, 104]. Interestingly, CD34^−^CD31^−^ ADSCs are also shown to differentiate into ECs and contribute to neovascularization, suggesting a common ancestor for a phenotypically different subpopulation of ADSCs [105].

*(3) Cardiac Tissue*. The perception that the adult heart is a postmitotic organ without regenerative capacity has changed dramatically after the isolation of C-KIT^+^Lin^−^ cells from the heart of adult rats [106]. These cells were shown to be self-renewing, clonogenic, and multipotent, exhibiting cardiogenic differentiation potential into three main cell types: cardiomyocytes (CMs), smooth muscle cells (SMCs), and ECs [106], which represent the cardiogenic lineage. When these C-KIT^+^Lin^−^ cells were injected into the ischaemic hearts of rats, functional myocardium was regenerated and more animal survived [106]. In contrast, another study demonstrated that SCA-1^+^C-KIT^−^ cells from mouse hearts could be induced by 5-azacytidine *in vitro* to differentiate towards the cardiac myogenic lineage. Furthermore, when administered intravenously, SCA-1^+^C-KIT^−^ cells ameliorated myocardial injury by differentiating into CMs [107]. Unlike C-KIT, SCA-1 is not expressed in humans. The first evidence of human cardiac stem cells (hCSCs) was found by isolating C-KIT^+^ cells from myocardial samples and observing that these C-KIT^+^ cells differentiated predominantly into CMs and, to a lesser extent, into SMCs and ECs. When these C-KIT^+^ cells were injected into the infarcted myocardium of immunodeficient mice and immunosuppressed rats, they could generate a chimeric heart containing human myocardium composed of myocytes, coronary resistance arterioles, and capillaries [108]. However, in recent studies by lineage tracing analysis in murine models, it was found that C-KIT^+^ cells generated significant numbers of ECs but insignificant numbers of CMs [109, 110]. Therefore, although research on the ideal signature of cardiac stem cells (CSCs) continues [111], it is pertinent to note that ECs are a component of the triad of the cardiogenic lineage (CMs, SMCs, and ECs). Whether the EC lineage is analogous to the haematopoietic lineage that gives rise to all blood cells or follows distinct pathways requires further research. {For ongoing research in cardiac stem cells and myocardial regeneration, which is beyond the scope of this review, readers can consider reviewing articles [111, 112].}

*(4) Neural Tissue*. Neuronal cells were thought to be tissue specific and unipotent until the transdifferentiation of adult neuronal stem cells (NSCs) into HSCs was demonstrated following their transplantation into the haematopoietic niche of mice [113]. However, the first evidence that neuroangiogenesis is a closely related phenomenon was uncovered by studying the fate of neuronal cells present in the subgranular zone (SGZ) of adult rats. During the first two hours after injecting BrdU- (bromodeoxyuridine-) labelled nonmitotic cells into the hippocampus of adult rats, approximately 37% of these cells exhibited endothelial markers, which gradually decreased to 9% after one month when more than 90% of the transplanted cells exhibited neural markers [114]. In fact, NSCs from the human embryo have also been shown to express several endothelial and haematopoietic markers [115]. *In vitro* studies supported that both NSCs [116, 117] and peripheral nerve-derived adult pluripotent stem cells (NEDAPS) [118] can be transdifferentiated to ECs. When NSCs were cocultured with ECs, only 6% of the NSCs differentiated into ECs. These differentiated ECs do not show any neurological markers; instead, they phenotypically express markers of the endothelial lineage [119]. Studies using an *in vivo* mouse model have shown that NSCs contribute to both neurogenesis and vasculogenesis in not only neuronal tissue but also nonneural tissue in adults [117]. All the above studies reflect two important findings: first, both NSCs and ECs share a common progenitor, and second, the local environment is crucial in governing the transdifferentiation of NSCs to ECs.

*(5) Dental Tissues*. Various types of stem cells have been found in the teeth and are referred to as dental stem cells [120].

*(5.1) Dental Pulp Stem Cells (DPSCs)*. Stem cells from dental tissue were initially isolated from the dental pulp and are termed as DPSCs. When DPSCs differentiated into osteogenic progenitors, some of the cells exhibit EC-like phenotypes and, therefore, gave rise to both osteoblasts and endotheliocytes [121]. This observation was further confirmed by another study showing that DPSCs form capillary-like structures in the presence of VEGFR and form vascular tubes on Matrigel, thereby suggesting their potential for EC differentiation [122].

Furthermore, when these DPSCs were transplanted *in vivo*, in the form of either woven bone tissue explants [121] or implanted as such in the chicken chorioallantoic membrane (CAM) assay [123], a significant increase in blood vessel density and infiltration within the host tissues was observed. In a recent study, it was also substantiated that these DPSCs can be used without any other ECs for regenerative purposes [124]. Moreover, the angiogenic potential of DPSCs was significantly increased when they were cocultured with ECs [125].

Another unique subset of stem cells referred to as side population cells (SPCs) has been identified in DPSCs. These SPCs are similar to EPCs as they express VEGFR-2 but not haematopoietic markers, such as CD45; however, unlike EPCs, SPCs do not express CD31 and CD146. SPCs also show multilineage differentiation potential and can be induced to form ECs with the expression of VEGFR-1^+^ and VEGFR-2^+^ and capacity to uptake acetylated-LDL and form a capillary-like network [126]. Furthermore, *in vivo* transplantation of SPCs can promote neovascularization of ischaemic mouse hind limbs [126, 127].

*(5.2) Stem Cells from Human Exfoliated Deciduous Teeth (SHED)*. Human deciduous teeth are excellent and most readily available sources of stem cells [128]. SHED can differentiate into ECs and form functional blood vessels that anastomose with the host vasculature [129, 130]. Although there was no significant difference in the number of new blood vessels formed by SHED alone or SHED in combination with human dermal microvascular ECs (HDMECs), the microvascular organization was significantly improved when SHED were cocultured with HDMECs, thus increasing the chances of survival after transplantation [129]. The mechanism by which SHED differentiate into ECs depends on the crosstalk between STAT3 and MEK-1/ERK signalling. Unstimulated SHED express high levels of phosphorylated STAT3, which has an inverse relationship with the *MEK-1/ERK* gene. Inhibition of STAT3 activity by VEGFR induces MEK-1/ERK phosphorylation, resulting in differentiation of SHED into ECs, whereas inhibition of *MEK-1/ERK* gene results in the maintenance of STAT3 activity or the stemness of SHED [130].

*(5.3) Stem Cells from Apical Papilla (SCAP)*. When SCAP and DPSCs were cocultured with ECs, the proangiogenic effect of SCAP was significantly weaker than that of DPSCs. In particular, although EC proliferation was not observed, significantly greater endothelial migration and tubulogenesis were noticed in DPSCs than in SCAP. Furthermore, both SCAP and DPSCs promoted angiogenesis in the CAM assay [131].

*(5.4) Periodontal Ligament Stem Cells (PDLSCs)*. Similar to MSCs, PDLSCs have been shown to produce more VEGFR than SHED. *In vitro* Matrigel assay has shown no significant difference in the number of blood vessels formed from PDLSCs, MSCs, and SHED [132]. In coculture experiments, the angiogenic potential of PDLSCs cultured with ECs was significantly enhanced [132, 133] compared to ECs alone, which is similar to the above-discussed dental stem cells.

In summary, EPCs are either housed (e.g., in bone marrow, peripheral blood, and umbilical cord blood) or produced by transdifferentiation from various sources under the influence of microenvironments suitable for endothelial differentiation (Figure 3). The four phases of neovascularization include differentiation, proliferation, migration, and attachment of EPCs to form tubes. In general, the above studies reflected that both housed and transdifferentiated EPCs act via a paracrine mechanism; however, proliferation is significantly higher in housed EPCs than in transdifferentiated EPCs.

## 6. Role of the Surface Markers in ECs

**Figure 5:**
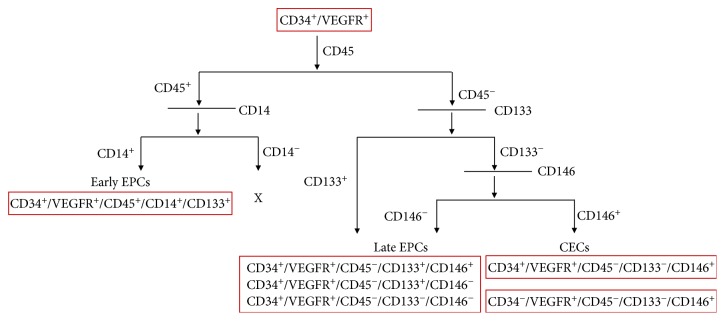
Phenotypic identity of ECs.

Cell surface markers are proteins and carbohydrates attached to the cell membrane which play an important role in identification and investigation of cells by providing a specific target. In brief, these cell surface markers are like a fingerprint, specific to each kind of cell and capable of being identified through immunophenotyping. Various types of cell markers have been identified in ECs, such as CD34, a haematopoietic stem cell marker which is present in all types of ECs [88, 134]. CD45, a pan leukocytic marker is present only on eEPCs and not on lEPCs or CECs [81, 88, 134]. AC133/CD133 which is expressed on both haematopoietic stem and progenitor cells (HSCs) [135] is also expressed on eEPCs, while on CECs it is absent, reflecting that it is an early marker. On the other hand, conflicting reports exist on the expression of CD133 by lEPCs [88, 89, 136]. CD14 is a monocytic lineage marker. Various studies have confirmed that CD14 is present on eEPCs but not on lEPCs and CECs [93, 137]. VEGFR-2 (Flk-1 in mouse or KDR in humans) is a prominent endothelial cell marker. eEPCs show weak expression (focal expression) of VEGFR-2, but VEGFR-2 is strongly expressed in lEPCs and CECs [81, 92, 93, 134, 137]. Human endothelium constitutively expressed CD146 (also referred to as MUC18, MCAM, Mel-CAM, S-Endo-1, or P1H12 antigen) irrespectively of the anatomical localization [138–140]. Therefore, CECs can be defined by their ability to express CD146. In fact, various studies were carried out to isolate and define CECs by the expression of CD146 [83, 85, 141], and then these CD146^+^ CEC cells were hypothesized to determine the prognosis of disease [142–144]. However, there is another caveat—CD146 can also be found on EPCs or even pericytes thereby further complicating the definitions. Additionally, various other markers, such as CD36, CD106, and vWf, have also been used infrequently in the literature. Therefore, in summary, a true definition of distinct ECs needs further investigation. Nevertheless, based upon the current evidence, we are here proposing the phenotypic definition of ECs which can apparently guide the researchers and scientific community in this field (Figure 5).

## 7. Current Prospects

The regulation of endothelial function by circulating EPCs opens a new avenue with immense potential in almost every realm of therapeutics because these circulating EPCs are affected by not only a plethora of exogenous and endogenous factors but also various pathological conditions. Therefore, EPCs are direct indicators of endothelial function.

### 7.1. Exogenous Factors

#### 7.1.1. Exercise

Exercise is considered to be indispensable and vital for maintaining normal physiological functions. It has been observed that exercise for 10 minutes increases the level of EPCs in circulation by up to four times [86]. At the same time, *in vivo* studies on mice and subsequently humans with stable coronary artery diseases have also found that exercise increases the level of EPCs [145]. The increased EPC count was due to the mobilization of EPCs which has been shown to be NO dependent in a knockout mouse model (eNOS^−/−^ mice) [145], wherein exercise enhances the phosphorylation of eNOS by activation of the PI3K (phosphatidylinositol 3 kinase/protein kinase B) pathway [146]. Further, it has also been demonstrated that hypoxia in tissues induced by exercise causes upregulation of hypoxia-inducible factor-1*α* (HIF-1*α*) which is responsible for the increased levels of proangiogenic cytokines, such as VEGF [147] and stromal-derived factor-1 (SDF) [148] with concomitant EPC mobilization.

Moreover, exercise also has a hormonal effect where it upregulated *β*2 adrenergic receptor signalling resulting in proliferation, migration, and differentiation by stimulation of proapoptosis and antiapoptosis pathways involving p38 MAPK (mitogen-activated protein kinases) and PI3K/AKT activation augmenting angiogenesis, both *in vitro* and *in vivo*, resulting in amelioration of neovascularization in animal models of hind limb ischaemia [149, 150].

It is also apparent through various observational and interventional studies that exercise helps in reducing inflammatory markers, such as C-reactive protein (CRP) and TNF-*α* [151]. CRP promotes apoptosis and attenuates the function and differentiation of EPCs [152], whereas TNF-*α* causes the diminution of proliferation and differentiation of EPCs [153]. As a result, exercise has a positive influence on EPCs by regulating inflammatory markers.

Subsequently, a positive correlation is established between exercise and EPCs in human subjects with acute myocardial infarction [154], chronic heart failure [155], peripheral arterial diseases [156], microvascular angina [157], and acute coronary syndrome [158] because all the above diseases have one common underlying pathogenesis, endothelial dysfunction, and reduced EPC number. Therefore, exercise is a major modifiable risk factor for cardiovascular disease (CVD) [159] where exercise plays a key role in attenuating the incidence and risk of CVD. Additionally, exercise is one of the components in the triad of “cardiac rehabilitation” (exercise counselling and training, education for heart-healthy living, and counselling to reduce stress) which according to American Heart Association (AHA) is a “medically supervised programme to improve cardiovascular health in case you have any experienced heart attack, heart failure, angioplasty, or heart surgery.” In fact, various studies have proven a beneficial effect on endothelial function by improving the number of EPCs [158, 160].

Hence, it can be acknowledged that a sedentary lifestyle is the root cause of many problems and exercise has a holistic effect on health and on EPCs through mobilization, proliferation, differentiation, function, and survival.

#### 7.1.2. Fasting

Fasting is an important ritual that is practised by many communities in the world. A certain degree of fasting may elicit profound and sustained beneficial metabolic, hormonal, and functional changes [161]. Recently, the effect of fasting on EPC regulation has been evaluated using a fasting stroke mouse model. In this study, focal cerebral ischaemia was induced in mice that subsequently underwent prolonged fasting (PF) or periodic PF. It was observed that PF not only significantly improved EPC-mediated angiogenesis, but also improved neurobehavioral outcomes. EPC functions, such as adhesion, migration and tube formation, as well as eNOS activity were significantly enhanced. Moreover, the volume of the atrophied brain and the size of the cerebral infarct were reduced compared to the control group. Furthermore, transplantation of EPCs from PF mice ameliorated the cerebral ischaemic injury in the same PF mouse models [162].

However, there is a difference in the effects of fasting between rodents and humans because it was found that in rodents, fasting decreases serum insulin growth factor-1 (IGF-1) concentration by approximately 30–40%, whereas in humans, fasting did not reduce total and free IGF-1 levels unless protein intake was also reduced [163]. Therefore, it is interesting to identify the cumulative effect of fasting on human EPCs because it is known that IGF-1 causes proliferation of EPCs via the PI3K/AKT signalling pathway [164].

#### 7.1.3. Smoking

Smoking is hazardous to human health and is a major risk factor for many diseases. Moreover, smoking has been shown to significantly impair endothelial function and integrity [165, 166] as well as the number of circulating EPCs [165]. Subsequently, other studies have also confirmed that the number of EPCs is reduced in chronic smokers and cessation of smoking leads to restoration of the normal EPC level [167, 168].

It is intriguing to note that nicotine, a primary addictive agent in cigarettes, is considered to have a beneficial effect at low concentrations but concentrations above 10^−6^ mol/L are cytotoxic [169]. Nicotine increases EPC number and enhances EPC proliferation, migration, adhesion, and vasculogenesis *in vitro* in a dose-dependent manner with maximum activity peaking at concentrations equivalent to 10^−8^ mol/L [169]. A study investigated the impact of nicotine on EPCs in an *in vivo* murine model where it was found that EPC counts were not only significantly increased when nicotine was administered for three weeks, but when nicotine was administered in ischaemic hind limbs for four weeks, there was a significant improvement in blood perfusion compared to controls. It was hypothesized that the increased EPC activity was due to the antiapoptotic effect of nicotine on EPCs via activation of the nicotinic acetylcholine receptor (nAChR) [170]. However, in another study, it was postulated that nicotine activates telomerase activity through either the PI3K/AKT pathway (by increased phosphorylation of human telomerase reverse transcriptase (hTERT) [171, 172] or upregulation of sirtuin type 1 (SIRT1) protein expression [172]. Telomerase activity may be responsible for cellular senescence [173]. Therefore, the increased proliferative capacity of EPCs is due to the prevention of cellular senescence by nicotine. It is important to note that the above conclusions were drawn from short-term studies evaluating the effect of nicotine on EPCs after nicotine exposure of fewer than four weeks.

Recently, the effect of nicotine exposure on EPCs was evaluated for six months. It was observed that short-term nicotine exposure increased the proliferative capacity of EPCs, which is consistent with the above-described studies. However, at six months, there was a marked reversal in the number, functional impairment, and telomerase activity of EPCs [172].

Therefore, nicotine exposure causes an increase in EPC count for one month. After that, prolonged exposure results in decreased EPC number. It is noteworthy that the increase in EPCs during the first four weeks does not warrant that smoking is good because tobacco smoke has >4000 chemical constituents of which majority of them are deleterious to ECs [174]. Thus, the net effect of smoking is detrimental to ECs. Instead, the results validated the previous study conducted almost seven years back which found that nicotine patches (as a part of smoking cessation therapy) in patients had a significant reduction in exercise-induced myocardial ischaemia [175]. On the other hand, the second statement, i.e., “prolonged exposure to nicotine resulted in decreased EPC number,” is an affirmation that smoking is injurious to health. As a result, the above study [172] underscores the cessation of smoking to prevent endothelial dysregulation.

#### 7.1.4. Psychosocial Factors

Depression is a common illness that has a negative impact on one's health and society [176]. It was observed that the number of mature and immature EPCs was reduced in patients with depression [177]. Furthermore, there was a significant inverse relationship between EPC levels and the severity of depressive symptoms [177].

In depression, the reduced EPC count is related to the increase in plasma concentrations of TNF-*α* and CRP [178]. CRP promotes apoptosis and attenuates the function and differentiation of EPCs [152], whereas TNF-*α* reduces the number of EPCs [153]. However, only TNF-*α* showed a statistically significant inverse correlation with EPC counts [177].

A study was conducted on healthy subjects (with no symptoms of angina or a history of CVD or diabetes mellitus) to explore the possible effect of depression on brachial artery flow-mediated dilation (FMD) and EPCs. It was found that depression was an independent predictor of decreased brachial FMD. Furthermore, impaired FMD was ultimately related to low levels of circulating EPCs [179]. Another study also reported similar results in patients with stable angina [180].

Therefore, endothelial dysregulation is also associated with depression.

### 7.2. Endogenous Factors

#### 7.2.1. Serum Cholesterol

Numerous studies have described the relationship of EPCs with lipid metabolism. Increased cholesterol level or hypercholesterolemia (HC) is one of the established risk factors for atherosclerotic vascular disease. It has been reported that HC attenuates angiogenesis and collateral vessel formation [181, 182]. Oxidized low-density lipoproteins (ox-LDL) induce dephosphorylation of the AKT kinase at Ser^473^ by activation of a serine/threonine phosphatase, resulting in deactivation of a downstream pathway or dephosphorylation of phosphorylated products of PI3K by either phosphatase and tensin homologue deleted on chromosome 10 (*PTEN*) or SH2-domain-containing inositol 5-phosphatase-2 (*SHIP-2*) [183]. PI3K is at the topmost of the endothelial regulation pathway and is composed of 2 protein subunits, p83 and p110. Both of these protein subunits must remain together for downstream activity [184]. However, ox-LDL causes nitrosylation of the P85 subunit, thereby impairing PI3K function and inactivating downstream pathways [185]. It is also important to note that the PI3K pathway functions in close association with the p38 MAPK pathway. PI3K induces cytoprotective effects, whereas the p38 MAPK pathway has proapoptotic effects. Therefore, dysregulation of the PI3K pathway by ox-LDL causes upregulation of the MAPK pathway, resulting in EPC apoptosis [186].

ox-LDL also causes upregulation of LOX-1 receptor expression in ECs, resulting in downregulation of eNOS expression and activity followed by AKT dephosphorylation [187]. The above findings are further confirmed by using mice genetically deficient in AKT [188] or eNOS [189], in which EPC function is reduced and postischaemic angiogenesis is compromised.

However, administration of VEGFR [181] or L-arginine [182] has been shown to augment angiogenesis in hypercholesterolemia partially. L-Arginine is the substrate for eNOS which in turn is responsible for the production of nitric oxide and plays a crucial role in the proliferation, migration, and delayed senescence of EPCs [190]. On the other hand, VEGFR or statins (HMG-CoA reductase inhibitors) induce EPC differentiation by stimulating the PI3K/AKT pathway [191]. Further, VEGFR also stimulates the p38 MAPK pathway. Therefore, the level of either p38 MAPK or PI3K activation decides whether EPCs undergo apoptosis or cell proliferation, respectively [192].

In summary, HC and, in particular, ox-LDL have a marked impact on the functional characteristics of EPCs, including proliferation, migration, and apoptosis.

### 7.3. Pathological Diseases/Conditions

#### 7.3.1. Hypertension

Chronic hypertension (CH) is one of the most prevalent diseases worldwide. Studies have shown that hypertension has an adverse effect on various stages of EPC regulation. For example, functional impairment (reduced mobilization) in EPCs is the most significant independent predictor of CH [95]. Furthermore, the levels of EPCs are significantly reduced in patients with hypertension. However, functional decline in EPCs seems to occur more commonly and earlier than the reduction in EPC quantity [193, 194] and lEPCs exhibit more significant declines in proliferative activity than other types of EPCs [195]. The relationship between hypertension and EPC function is further strengthened by the effect of antihypertensive drugs on EPCs through their different mechanisms of action [196–199] {readers can read the review by Luo et al. [200] to acquire more detailed insight on matters beyond the scope of this review}.

#### 7.3.2. Diabetes Mellitus

Diabetes mellitus is a metabolic anomaly characterized by increased glucose intolerance. Patients with type 1 and type 2 diabetes have fewer circulating EPCs compared to matched healthy subjects because of the reduced mobilization of EPCs from bone marrow either due to insufficient release of marrow-stimulating factors, such as VEGFR and SDF-1, which resulted in downregulation of hypoxia-induced factor (HIF-1) [201] or through the PI3K-AKT-eNOS pathway [190, 191]. Moreover, EPCs of diabetic patients exhibit reduced proliferation, adhesion, migration, and incorporation into tubular structures [202, 203]. Additionally, there is an increase in EPC apoptosis due to the upregulation of ROS (reactive oxygen species) caused by hyperglycaemia and oxidative stress [204–206]. Therefore, diabetes affects all EPC regulatory pathways.

Also, many complications of diabetes, such as diabetic vasculopathy, cardiomyopathy, neuropathy, nephropathy, and retinopathy, are closely linked to the problem of vascularization [207]. Interestingly, among all these complications, there is a marked reduction in EPCs, except for retinopathy, which follows a reverse pattern [207]. Hence, diabetes is an “angiogenic paradox” in which the same diabetic patient at the same time can present with the complications of pervasive angiogenesis (for example, diabetic retinopathy) and of diminished angiogenesis (for example, symptomatic peripheral arterial disease (PAD) in diabetic vasculopathy). It is further convoluted that the integration of different complications might result in different outcomes from the one which is expected. For example, diabetic foot syndrome (DFS) can be due to the combination of neuropathy and PAD, and therefore, presumably, there should be a reduction in EPCs. However, it was found that the number of circulating EPCs in patients with diabetes and manifesting DFS was higher than that in patients with uncomplicated diabetes [208]. A positive correlation of VEGF-A, a proinflammatory cytokine, has been found to be associated with EPCs in diabetic patients [208, 209]. It has been postulated that the ischaemic tissues are responsible for the elevated levels of VEGF-A which in turn is responsible for the increase in circulating EPCs [208, 209]. Hence, comprehending the regulatory mechanism of angiogenesis and their association with EPCs might lead to EPC-based therapies, a clinical reality in treating diabetes.

#### 7.3.3. Cardiovascular Diseases

Endothelial dysfunction has been shown to be closely associated with CVDs, such as coronary artery disease (CAD), myocardial infarction (MI), and ischaemia [176]. The correlation was first reported by Shintani et al., where they identified that the CD34^+^ cells did not differ between the MI patients and controls on day 1 but CD34^+^ cell levels appeared to linearly grow, reaching a peak after seven days with a statistically significant difference as compared to controls [210]. In sharp contrast, another study found that the number of CD34^+^ cells was significantly higher in patients with MI at admission than in controls. This study even showed a decreasing trend in the number of circulating CD34^+^ cells in patients with MI, with the number of CD34^+^ cells significantly lower on day seven than at admission but still higher than those in control patients [211]. It is noticeable that in the first study, authors presumed that EPCs originate from CD34^+^ cells and did not quantify EPCs and, instead, performed a cell culture assay and stated that EPCs and their putative precursor, MNC CD34^+^, were mobilized into PB during an acute ischaemic event in humans peaking at 7 days [210]. However, in another study, authors quantify EPCs which were at a higher level at admission than at day 7 [211]. It is also interesting to note that the level of VEGF is proportional to that of CD34^+^ in both studies [210, 211], but the VEGF level in first study peaked at day 7 [210], while in another study, it was highest at day 0 [211]. Nevertheless, in both studies, release of VEGF from ischaemic tissues was implicated as the primary factor responsible for increased EPCs.

In cases of chronic ischaemic cardiomyopathy, there was no significant difference in the number of progenitor cells between chronic ischaemic cardiomyopathic patients and controls, except in *in vitro* studies, where the functional capacity of EPCs (evaluated as colony-forming activity and the migratory response) appeared to be significantly reduced in chronic ischaemia patients as compared to controls [212]. However, patients with unstable angina had a significantly greater number of circulating EPCs and EPC-CFUs than patients with stable angina [213]. In this study, the authors discovered a positive correlation between CRP levels and EPC levels. However, they did not notice any interaction between EPCs and VEGF.

To elucidate the role of EPCs in CVD, preclinical pig or rat models with ischaemic and infarct conditions were used [214, 215]. EPCs improved cardiomyocyte survival, increased myocardial contractility, and decreased the infarct size. Furthermore, thymosin *β*4, which is an essential paracrine factor of EPCs, also ameliorated the prognosis of myocardial infarction by reducing cardiomyocyte apoptosis, increasing myocardial contractility and decreasing the infarct size [214, 215]. Therefore, EPCs not only are considered a prognostic marker but also are of therapeutic value in CVD.

#### 7.3.4. Cerebrovascular Diseases

There is a well-established relationship between cerebrovascular disease and EPCs. The number of circulating EPCs has been shown to increase rapidly in the acute phase of ischaemic stroke [216–219]. The increase in EPCs has been associated with positive neurological and functional outcomes, reduced infarct growth, and neurological improvement [219–221].

The most severe complication of stroke is intracerebral haemorrhage (ICH), which occurs in approximately 10–15% of all stroke cases. In a recent study on patients who had suffered from an acute ischaemic stroke, increased EPC count at day seven is associated with good functional outcome and reduced ICH residual volume [222]. With limited therapeutic options for ICH, studies on EPCs are important for the development of new treatment modalities.

Additionally, the fate of EPCs after transplantation into areas of ICH needs to be explored further.

#### 7.3.5. Erectile Dysfunction

Erectile dysfunction is defined as the consistent inability to obtain or maintain an erection for satisfactory sexual relations. There are two types of erectile dysfunction: vasculogenic and neurogenic erectile dysfunctions. As endothelial dysfunction is considered one of the aetiologies of erectile dysfunction, the relationship between vasculogenic erectile dysfunction and EPCs is of interest. The number of circulating CD34^+^CD133^+^ EPCs is significantly reduced in patients with erectile dysfunction without known cardiovascular risk factors [223]. In patients with cardiovascular risk factors, although the number of CD34^+^/VEGFR-2^+^ cells is not affected, the number of CD133^+^ circulating EPCs is reduced [224]. Furthermore, as erectile dysfunction is also a complication of overweight and type-1 DM, the number of CD34^+^VEGFR-2^+^ EPCs has been found to be correlated with the severity of erectile dysfunction [225, 226]. Therefore, EPCs might serve as a valuable diagnostic tool.

Regarding treatment strategies, a recent study showed that intracavernous injection of EPCs into the corpora cavernosa of rats with erectile dysfunction caused by bilateral cavernous nerve injury could restore erectile function [227]. In summary, the close correlation between erectile dysfunction and penile vascular dysfunction suggests that EPCs may have great therapeutic potential.

#### 7.3.6. Cancer

Vascularization is a critical component of tumour growth and progression. Moreover, EPCs (including CECs) are increased in the peripheral blood of patients with various cancers, such as multiple myeloma [228], acute myeloid leukaemia [229, 230], nonsmall cell lung cancer (NSCLC) [231], hepatocellular carcinoma [232, 233], breast cancer [234–236], ovarian cancer [237], chronic lymphocytic leukaemia (CLL) [238], renal cell carcinoma (RCC) [239, 240], and endometrial cancer [241]. CECs have been implicated in tumour progression and aggressiveness [230, 231, 235, 237, 238, 240, 241]. Furthermore, in malignant breast carcinoma, EPCs are resistant to the cytokine TNF-*α*, which is responsible for inducing apoptosis [242].

Therefore, in cancer, EPCs may not only serve as a biomarker but also their regulation may be a critical therapeutic approach.

## 8. Current Clinical Trials

As EPCs play a significant role in pathophysiological functions of the human body, EPC therapies for various cardiovascular, endocrine, haematological, renal, respiratory, neoplastic, and other diseases are underway. A summary of clinical trials is shown in Tables 2 and 3. The data were taken from ClinicalTrials.gov after inputting EPCs as a “*key word*.” A total of 302 trials were registered as of 30/1/2018. Out of these 302 trials, 45.36% are completed and the remaining studies are in different phases (Table 2). Furthermore, it was intriguing to find that more than one-fourth of the studies were terminated, were withdrawn, or had unknown status (Table 2). It was also noteworthy that most clinical trials studied the relationship between EPCs and CVDs followed by endocrinal disorders and other diseases (Table 3). However, only one study focused on isolating dental mesenchymal cells from impacted teeth to construct prevascularized tissue-engineered bone.

## 9. Future Prospects

EPCs will play a pivotal role in regenerative medicine and cancer therapy besides acting as surrogate markers of future health problems. However, collaborations among clinicians, biomaterial scientists, and engineers will be pertinent to resolve various issues and to enable quick clinical translation.

### 9.1. Novel Stem Cell Differentiation and Animal Models

Further exploration of molecular and cellular events underlying the regulation of EPCs using newer stem cell differentiation and animal models is the need of the hour.

### 9.2. Isolation and Consensus on the Identity of EPCs

Significant progress has been made in cytology, but a novel EPC marker still needs to be identified. Furthermore, it is difficult to form a conclusion from results acquired using different protocols and apply the information to future advancements/clinical trials. Therefore, when expanding the knowledge, standardizing the identification of EPCs by both phenotype and function is imperative.

### 9.3. Rarity of EPCs

The number of EPCs in either peripheral blood (0.01%) or bone marrow (0.05%) is low; therefore, it is notoriously difficult to isolate EPCs. New advances in EPC isolation methods are required to improve success and yield.

### 9.4. Expansion of EPCs

Irrespective of the source, after the isolation of EPCs, the number of cells must be increased before further applications. However, passaging will inadvertently shift stem cells towards maturity with diminished stemness. Currently, methods to increase EPC number without increasing the passage of cells are lacking.

### 9.5. EPC Homing and Incorporation

Homing of EPCs will enable the targeted delivery of EPCs to the site of interest. With recent advances in nanotechnology and tissue engineering, the local distribution of cells seems to be possible.

### 9.6. Modulation of the Host Environment

It is agreed that cell survival and function depend on the local or systemic environment of the host. Hypoxia, increased inflammation, and free radicals may have adverse effects on EPC survival. Therefore, modulation of the host environment is very crucial to the success of cell-based therapies.

### 9.7. Translation of Bench-Side Models

Emphasis should be given to the translation of bench-side EPC study models to clinical trials.

## 10. Summary and Conclusion

With the identification of EPCs, the domain of neovascularization has metamorphosed. The findings from various research studies have begun to coalesce like a jigsaw puzzle. With significant achievements over a century, the origin of EPCs, the role of EPCs in angiogenesis and the physiopathological process, and the potential EPC-based therapeutic approaches have begun to be uncovered, but still, a lot of work remains. Clinically, EPCs can be applied in three different ways:

1. *Potential Biomarker*. Disease identification and severity
2. *Target Cells*. Anti-EPC therapy for tumours/cancer
3. *Neovascularization*. Either alone or cocultured with various stem cells

Although the complexity surrounding the biology of EPCs has increased, the comprehensive understanding of EPCs has also increased; therefore, EPC-based therapies may eventually become a clinical reality.

## Figures and Tables

**Table 1:** Differences between eEPCs and lEPCs.

	Early EPCs	Late EPCs
Synonyms	CACs [82]	OECs [83, 91] or ECFCs [84]
Cell population [81, 91]	Heterogeneous	Homogenous
Cell morphology [81]	Spindle-shaped cells	Cobblestone-like cells
Appearance in culture	<1 week [5, 81]	2–4 weeks [81]
Lifespan [81]	3-4 weeks	≈12 weeks
Morphogenic potential [81]	Low	High
Angiogenic potential [81]	Good	Good
Tube formation *in vitro* [5, 92, 134]		
*Tube formation by EPCs alone*	*Absent*	*Present*
*Tube formation by EPCs with HUVECs*	*Absent*	*Present*
Tube formation *in vivo* [92]	Absent	Present
Neovascularization *in vivo* [82, 92, 93, 134]	Indirect paracrine fashion	Directly providing ECs; hence can be referred to as “true EPCs”
*Surface expression*		
CD34 [88, 134]	*+*	*+*
CD45 [81, 88, 134]	*+*	−
CD14	+ and − [91, 93]/+ [92]	−
CD133	− [88] and + [89, 136]	− [88, 89, 136]
CD31 (PECAM 1) [81, 134, 137]	*−/+*	*++*
VEGFR-2 [81, 92, 93, 134, 137]	*−/+*	*++*
VE cadherin [81, 93, 134]	*−/+*	*++*
vWf [81]	*−/+*	*+*
Phenotype [90]	Monocytic	Endothelial
AcLDL uptake [5]	+	++
Lectin binding [5]	+	++
NO production [5]	+	++

+: present; ++: strongly present; −: absent; and −/+: limited/weak/focal.

**Table 2:** Current status of EPC clinical trials related to various disorders and diseases. The table outlines the total number of clinical trials reported on ClinicalTrials.gov till 30/1/2018.

	Completed	Recruiting	Active, but not recruiting	Not yet recruiting	Terminated	Withdrawn	Unknown status	NA	Enrolled by invitation
Single parameter of a disease or disorder	90	37	13	8	14	10	38	0	1
Different parameters in a disease or combination of diseases with single or multiple parameters in each disease	47	17	3	3	7	1	12	1	0
*Total * **(302)**	**137**	**54**	**16**	**11**	**21**	**11**	**50**	**1**	**1**

**Table 3:** Current status of clinical trials investigating EPCs and its relation to a single factor in disorders or diseases.

	Completed (90)	Recruiting (37)	Active, but not recruiting (13)	Not yet recruiting (8)	Terminated (14)	Withdrawn (10)	Unknown status (38)	NA (0)	Enrolled by invitation (1)
Disorder of CVS	47	15	7	1	8	5	15	0	
	e.g., MI, angina, hypertension, peripheral vascular disease (PVD), arteriosclerosis, coronary artery ischaemia	e.g., MI, cardiomyopathy, coronary artery disease (CAD), PVD, critical limb ischaemia (CLI), atherosclerosis	e.g., CAD, CLI, atherosclerosis	CLI	e.g., angina, CAD	e.g., ischaemic congestive heart failure (CHF), lower limb ischaemia	e.g., aortic aneurysm, CHF, CAD, PVD		

Renal disease	2	1							
	End-stage renal disease and chronic kidney disease	Acute kidney injury							

GIT disorders	3	2					1		
	Liver cirrhosis, nonalcoholic fatty liver disease, and severe hepatic venoocclusive disease	End-stage liver disease and sinusoidal obstruction syndrome					Crohn's disease		

Endocrine disorders	13	4	2	2		2	5		
	e.g., type 1 and type II DM	e.g., type II DM	e.g., type II DM etc.	e.g., type II DM etc.		e.g., type II DM	e.g., type 1 and type II DM		

Neoplastic disorders	3	2			4		5		
	e.g., breast cancer, colorectal cancer, and nonsmall cell lung cancer	e.g., BRCA1, BRCA2 gene mutation, cervical cancer			e.g., renal cell carcinoma, multiple myeloma		e.g., breast cancer, lung cancer		

Healthy subjects	7	1	1	1			1		
	e.g., healthy subjects	Obesity	Healthy	Quality of life			Morbid obesity		

Respiratory disease	2	1		1			3		
	Idiopathic pulmonary arterial hypertension	Pulmonary emphysema		Pulmonary hypertension			e.g., chronic obstructive pulmonary disease (COPD), idiopathic pulmonary arterial hypertension		

Reproductive disorders	3				1				
	e.g., polycystic ovary syndrome (PCOS)				e.g., PCOS				

Musculoskeletal disorders	1		1						
	Ankylosing spondylitis		Bone defects						

Neurologic diseases	2	4	1			2	2		1
	Central nervous system and acute ischaemic stroke	Stroke, aneurysmal subarachnoid haemorrhage, and mild cognitive impairment	Brain and central nervous system tumours			Traumatic brain injury	Ischaemic stroke and migraine with aura		Diabetic foot ulcer

Hematological disorders	4	5		2	1	1	4		
	Endotoxemia, sickle cell anaemia, dyslipidemia, and hypoxia	Sickle cell disease without crisis, hypercholesterolemia, microgravity-exposed endothelial cells, hemolytic uremic syndrome, and soft-tissue sarcoma		Dystrophic epidermolysis bullosa and septic shock	Graft-versus-host disease	Sickle cell anaemia	Exercise anaphylaxis, recurrent adult Hodgkin lymphoma, acute myeloid leukemia, and brachial plexus (pressure)		

Autoimmune disorders		1					1		
		Systemic scleroderma					Systemic lupus erythematosus		

Dental disorders				1					
				Tooth impacted					

Miscellaneous	3	1	1				1		
	Burn, bullous keratopathy, ischaemic ulcer	Sepsis	Delayed graft function				SDF-1		
